# Process-driven and biological characterisation and mapping of seabed habitats sensitive to trawling

**DOI:** 10.1371/journal.pone.0184486

**Published:** 2017-10-05

**Authors:** Aurélie Foveau, Sandrine Vaz, Nicolas Desroy, Vladimir E. Kostylev

**Affiliations:** 1 Laboratoire Environnement et Ressources Bretagne Nord, Ifremer, Dinard, France; 2 UMR MARBEC, Ifremer, Sète, France; 3 Geological Survey of Canada (Atlantic), Natural Resources Canada, Dartmouth, Nova Scotia, Canada; University of Waikato, NEW ZEALAND

## Abstract

The increase of anthropogenic pressures on the marine environment together with the necessity of a sustainable management of marine living resources have underlined the need to map and model coastal environments, particularly for the purposes of spatial planning and for the implementation of integrated ecosystem-based management approach. The present study compares outputs of a process-driven benthic habitat sensitivity (PDS) model to the structure, composition and distribution of benthic invertebrates in the Eastern English Channel and southern part of the North Sea. Trawl disturbance indicators (TDI) computed from species biological traits and benthic community composition were produced from samples collected with a bottom trawl. The TDI was found to be highly correlated to the PDS further validating the latter’s purpose to identify natural process-driven pattern of sensitivity. PDS was found to reflect an environmental potential that may no longer be fully observable in the field and difference with *in situ* biological observations could be partially explained by the spatial distribution of fishery pressure on the seafloor. The management implication of these findings are discussed and we suggest that, used in conjunction with TDI approaches, PDS may help monitor management effort by evaluating the difference between the current state and the presumed optimal environmental status of marine benthic habitats.

## Introduction

**Fig 1:**
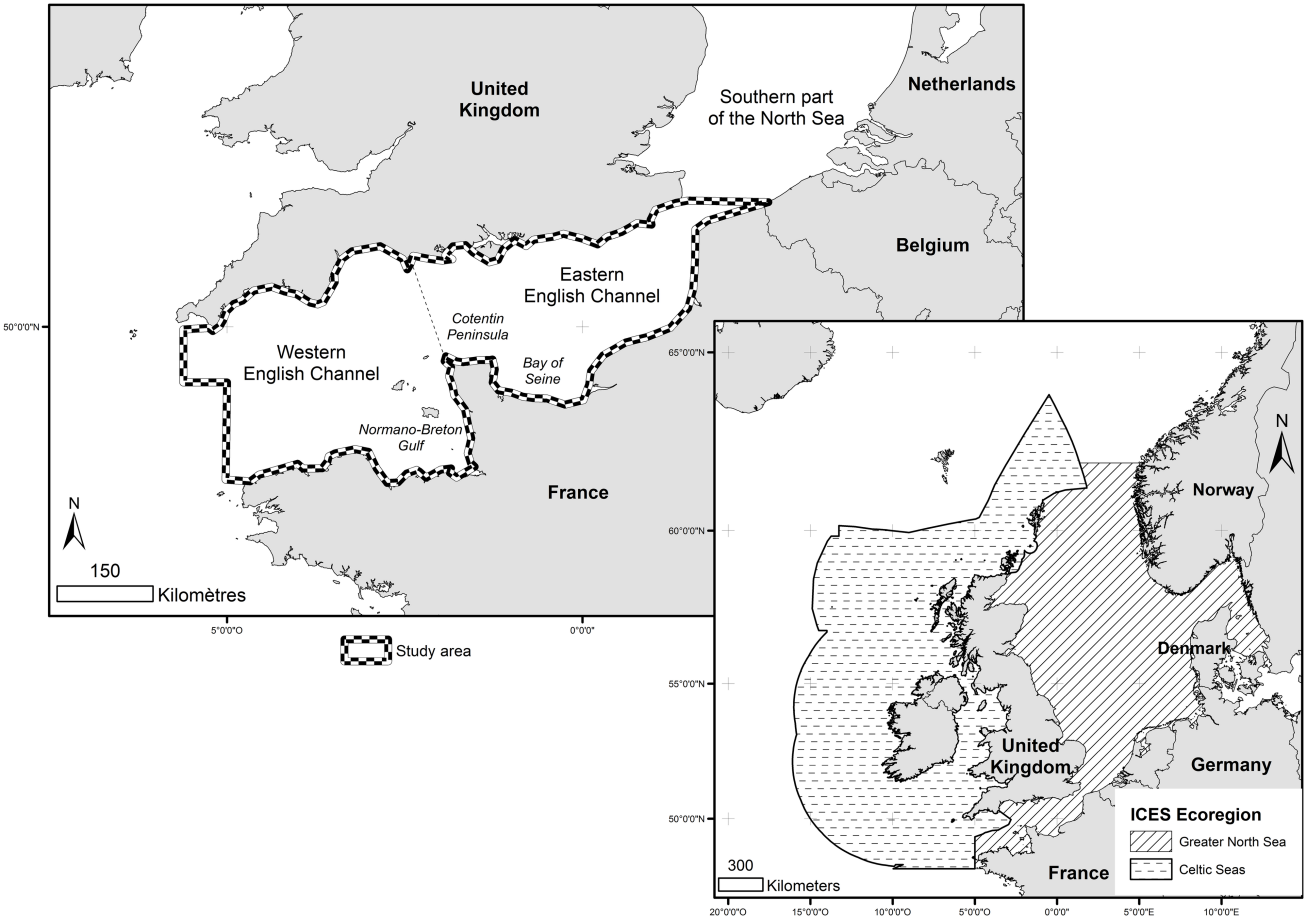
Localisation of the study area.

The Exclusive Economic Zone (EEZ) of the European Union (EU) represents (including overseas territories) an area of about 28 million km^2^, *i*.*e*. more than 20% of the World’s EEZ [1]. This important sea area offers a significant proportion of marine resources to each member state and contributes to economic prosperity and social well-being [2]. In order to protect this marine environment in the context of sustainable development [3], an integrated European marine policy has been developed. One of the main tools of the policy is the Marine Strategy Framework Directive (MSFD), adopted by the EU in July 2008 which aims to achieve a Good Environmental Status (GES) across Europe’s marine environment by 2020. In order to achieve this, four European marine regions were defined based on geographical and environmental criteria [4]. The North East Atlantic Marine Region was further divided into four sub-regions, and the English Channel was mainly included in the region called the “Greater North Sea” (Fig 1) [5]. Each member state is required to develop a marine strategy for their waters, based on (1) an initial assessment of the current environmental status of marine waters; (2) the adjusted definition of GES; (3) the designation of targets and indicators to achieve GES; (4) the definition of monitoring programs to measure progress or achievement of GES [4]. Eleven descriptors were assigned to monitor GES [6], some of which, such as seafloor integrity, are important to assess sensitivity of ecosystems to fishing and other types of human pressures and ensure that structure and functions of ecosystems are safeguarded and that benthic ecosystems, in particular, are not adversely affected.

Many definitions of ecosystem sensitivity have already been developed [7], [8], [9], [10], [11], [12], [13]. In all of these definitions, sensitivity is understood as (1) the vulnerability of an individual, a species, a population, a community or a habitat to an adverse impact of external factors, natural or anthropogenic (as opposite of stability); (2) the time necessary to return to the previous state (recoverability or resilience). As such, recoverability is a temporal term, dependant of processes like growth, recruitment or mortality while vulnerability is a state term, related to structures (*e*.*g*. body size or type) and functions such as mobility or defences. Sensitivity therefore encompasses both the strength of the effect of a particular impact (sometimes referred to as disturbance, perturbations or stress) on a receptor (*e*.*g*. species or habitats) and the recovery rate.

Process-driven seafloor habitat sensitivity (PDS) has been defined from the method developed by Kostylev and Hannah [14], which takes into account physical disturbances and availability of energy for growth and reproduction as structuring factors for benthic communities [15]. Kostylev and Hannah’s model is a conceptual model, relating species’ biological traits to environmental properties. The theoretical habitat template basis for this approach has already been formulated by Southwood [16] and established in multiple studies [17], [18], [19], [20], [21], [22]. In operational implementation, physical environment maps have been converted into a map of benthic habitat types, with assumption that each habitat type supports species communities with specific sensitivity to environmental pressures. For example, it is assumed that undisturbed low productivity habitats would more likely contain slowly growing and slowly reproducing sessile species, while highly disturbed habitats would be more suitable for mobile species with shorter life span and high reproduction rates [14]. This model was already applied in different geographic areas and with different biological groups (*e*.*g*. [14], [23] for Nova Scotia; [24] for Northern Spain; [25] for British Columbia; [26] for Canadian offshore; [27] for invertebrates in Alaska; [28] for North Atlantic fishes). This habitat modelling approach assimilates the scattered information on different physical variables (for example sediment grain size, seabed currents, mean water temperature) into a coherent two factors representation of seabed habitat types, in terms of relative values of seabed disturbance and scope for growth (theoretical amount of energy available for benthic growth and reproduction accounting for adaptation to adverse environment). *In situ* observations of benthic communities, however, remain necessary to validate and update the established models. In each particular application (*e*.*g*. new geographic region), this approach has to follow several steps: (1) identification of the environmental layers necessary for the construction of the model, (2) adjustment of these data sets, (3) model construction/adjustment following disturbance and scope for growth logic, (4) comparison of the model outputs with existing benthic communities and biological traits of key species and (5) comparison of model outputs with spatial distribution of human activities, having potential impacts on the environment (*e*.*g*. aggregate extraction, offshore wind farming, fisheries, for example). Because PDS outputs are based on idealised “natural” environment, we focus this paper on the comparison of the PDS results with other useful indicators of human impacts.

One of such indicators is a Trawl Disturbance Indicator (TDI), used on the European shelves, where intensive fishing has been undergoing for several decades over extensive areas [29], [30]. The indicator [31], [32], based on benthic species biological traits (mobility, fragility, position on substrata, average body size and feeding mode), was proposed for evaluating sensitivity of mega- and epifaunal communities to fishing activities impacting the seafloor (*e*.*g*. dredging and particularly bottom trawling). The selected biological traits were chosen because they determine individual vulnerability to trawling and they can be easily related to the fragility, recoverability and vulnerability concepts described earlier. Both the process-driven and biological traits approaches seem appropriate to investigate benthic habitat sensitivity. Moreover the differences between PDS, interpreted as a potential sensitivity descriptor, and TDI, which accounts for the abundance of sensitive species effectively observed, may be proposed as a helpful indicator to illustrate the distance that separates us from the good environmental status objectives.

The English Channel is suited to such an approach because of the abundance of data and variability of environment. It is a shallow epicontinental sea subjected to a megatidal regime that constitutes a biogeographical transition zone between the Atlantic Ocean and the North Sea [33]. Rich benthic communities have been described in the two basins of the Channel, however improved knowledge on the sensitivity of marine habitats is required to underpin the management advice provided for managers (*e*.*g*. on marine protected areas), as well as to support other marine monitoring and assessment work. This area is strongly impacted by human activities, whether traditional (fisheries, navigation, sediment disposal) or emergent (aggregate extraction, marine renewable energy) [34], [35]. In this study, the impact of fishing activities was tested as it reflects the presence of numerous commercial key fish species, nursery habitats, spawning areas or migration routes and is a historical activity structuring local communities and ecosystems.

Therefore, the present study aims to evaluate sensitivity of benthic habitats to trawling impacts by using the two approaches 1) a process-driven approach to predict the distribution of sensitive benthic communities (PDS), and 2) an index derived from *in situ* observations focusing on relevant biological traits (TDI). Additionally, we aim to account for the effect of recent bottom fishing impacts (evaluated as observed seabed abrasion by bottom trawling) to explain potential differences between approaches 1 and 2. The results are discussed in the frame of their usefulness to the MFSD GES objectives.

## Methods

### Study area

The study area encompasses the entire English Channel, from the southern bight of the North Sea to the Atlantic Ocean and which is divided into western and eastern basins (Fig 1) [35]. Water depth does not exceed 100 m, except in the northern North Sea (the Norwegian Trench) and near the Cotentin Peninsula [36], [37]. Water exchanges are principally driven by the inflow of oceanic Atlantic waters, via the English Channel. Bathymetry and coastlines of the Channel result in a high tidal range and complex megatidal regime and specific hydrodynamics conditions (existence of fronts and gyres), which control advection processes, dispersion of living organisms or pollutants [38], [39], [40], [41], [42], [43].

Hydrodynamics strongly influences sedimentation processes. The English Channel has limited sediment sources and extensive reworking of the sediment cover [37]. Seabed is considered as a submarine erosion surface, with fine sediments (silt and mud) located in bays and estuaries and in the southern part of the North Sea [44], [45] while coarse sediments (gravel) dominate in offshore areas of the Channel [39], [46], [47]. The distribution of habitats, benthic communities and species in the Channel is driven by depth, seabed substrate and water masses, with Western basin 50 m deeper than the Eastern, and although the two basins could be seen as distinct, they are not independent ecosystems [35]. As one of the world’s busiest straits for maritime shipping (accounting for 20% of global maritime traffic), the English Channel is also an overcrowded area subjected to strong anthropogenic pressures (resulting from fisheries, mineral extraction, offshore wind farms, pollution from maritime accidents, etc.) [48].

### Benthic habitat natural sensitivity: Axes of the model and risk map

The model developed by Kostylev and Hannah [14] is based on reducing multiple environmental variables into two explanatory axes (Disturbance and Scope for Growth) reflecting environmental forces that condition the presence of certain biological traits and hence sensitivity of benthic habitats and associated communities to environmental impacts.

The "Disturbance" (Dist) axis reflects the magnitude of change (alteration or destruction) of physical structure of habitats (i.e. the physical stability of habitats through time), due to natural processes influencing the seabed. In this paper, we estimated disturbance from current-, wave- and tide-induced sediment mobility as a ratio between the observed friction velocity and critical shear stress required to mobilise sediment particles. Environmental data layers were obtained from different sources, at different spatial and temporal resolution. The spatial resolution was standardised to a common grid of 0.03 x 0.03 decimal degrees, using resampling when required. The temporal resolutions were averaged or percentiles were used to illustrate the variables on a period of time that was relevant and closely matched those of the observed *in situ* biological data. The calculations were carried as follows.

The characteristic friction velocity was computed using the following data:

- depth: bathymetric data were obtained from the CHARM project, which were originally derived from SHOM navigation charts and transformed to raster with 1 km spatial resolution. Mean sea level data were obtained from MARS 3D hydrodynamic model and also transformed to raster (see [49]);
- waves: data were obtained from NORGAS model and compiled for years 2007 to 2009. The model has temporal resolution of 3 hours. Significant period and height data were calculated from the 90^th^ percentile [50];
- bottom currents: data were obtained from 3D MANGA model (Previmer, Ifremer) and compiled for years 2004 to 2007. The temporal resolution is one hour. Data were used to compute first the daily average, then the 90th percentile, of the bottom currents over the available period [50].

The detailed calculation steps for the wave generated current, which used depth and waves, are given in S1 File.

Finally characteristic friction velocity was estimated for each map grid cell as:

Frictionvelocity=[wavegeneratedcurrent]+[bottomcurrents] (1)

with wave generated currents calculated from the depth, peak wave period and wave height parameters and the tidal currents at the bottom were assumed to be collinear with wave generated currents.

The critical shear stress was computed as a function of the grain size (X = log_10_(grain size in mm) and derived from the Hjulström diagram [51] empirically approximated by the equation:

$$Y=\;-0.0272\;\times \;X^{4}-\;0.0905\;\times X^{3}+\;0.2411\;\times \;X^{2}+\;0.4691\times X+1.8761$$ (2)

Since hydrodynamic forces bring few changes to the overall sedimentary cover in the Channel [52], a mean grain size map for surficial sediments was obtained by assigning the observed mean grain size values from sediment samples [52] to each of the five sediment classes of a simplified sediment map but covering large geographical extent [39], [46].

The disturbance was then defined as the ratio between the intensity of friction (or characteristic friction velocity as defined in Eq 1) and the minimal current velocity needed to initiate sedimentary movement (critical shear stress defined as 10^Y^ with Y given by Eq 2).

The "Scope for Growth" (SfG) axis takes into account environmental stresses inducing a physiological cost to organisms and limiting their growth and reproduction potential. This axis estimates the remaining energy available for growth and reproduction of a species (the energy spent on adapting itself to the environment being already taken into account). The considerations in modelling SfG are conceptually related to the metabolic theory of ecology developed by Brown and collaborators [53]. In our implementation (*e*.*g*. [14]), SfG is not explicitly modelled as outcome of physiological processes in benthic communities, but is rather an evaluation of the benthic environment through relevant proxies. In assessing SfG, we have considered the following factors:

- food availability to benthic communities: calculated from monthly means of satellite-derived chlorophyll-*a* data, from 1998 to 2006[43]. Data were corrected using the equation of Legendre and Michaud [54] (log [POC] = 2.27 + 0.35 log[Chla], applicable for depth shallower than 300 m) to obtain values for Particular Organic Carbon, considered as food for benthic organisms;
- monthly mean bottom water temperature and its range of variability (inter- and intra-annual variations) were calculated using data from 2000 to 2006. Data were obtained from NOAA satellite-derived sea surface temperature dataset (see [49]);
- mean bottom water salinity was calculated from outputs of ECOMARS-3D model for years 2000 to 2006, monthly, with a temporal resolution of six hours;

High hydrodynamic energy and shallow waters result in intense mixing of the water column and absence of stratified waters in the most of the study area [55]; therefore, we assumed that surface temperature and chlorophyll concentration are correlated to bottom values and we used it as a proxy to food availability as described above. So, chosen variables are believed to be relevant to the construction of the SfG axis.

Before compiling the different environmental layers to define the SfG axis, data were rescaled between 0 and 1 to express their effects on a relative scale (following the next equation: $\frac{\left(Observed\;value\;of\;parameter-Parameter\;minimum\right)}{\left(Parameter\;maximum-Parameter\;minimum\right)}$). All grids were brought to the same spatial resolution. Since reclassification is sensitive to the geographical extent of the studied area (Fig 1), all layers were limited to the same spatial extent.

The SfG index was calculated as:

$$SfG=\;\frac{\left(F_{a}+SST+S-\;T_{a}-T_{i}\right)}{5}$$

where F_a_ is the food availability,; SST, the sea surface temperature; S, the salinity; T_a_, the standard deviation between monthly mean temperature within a year (calculated for each year and averaged over the study period) and T_i_ is the standard deviation of average annual temperatures between years

The SfG is an accumulative index, which allows characterization of the environment on a continuous scale between ‘benign’ (high Scope for Growth) and ‘adverse’ (low Scope for Growth).

Both disturbance and SfG indices were rescaled to 0–1 range.

The process-driven sensitivity (PDS) index corresponds to the inverse of distance from the origin of Dist-SfG quadrant, and can be visualised as a risk map that combines the two axes, according the formula:

$$Risk=\sqrt{(1-SfG)²+(1-Dist)²}$$

The PDS index assigns the highest sensitivity (and consequently the highest risk to benthic community) to habitats with low natural disturbance and low SfG. The combination of these axes was done using Raster Calculator in ArcGIS 9.2.sp6 with Spatial Analyst toolbox. From this point onward, the PDS term clustered the Disturbance, Scope for Growth and Risk term (these terms could be used independently if needed).

### *In situ* observation of benthic fauna

**Fig 2:**
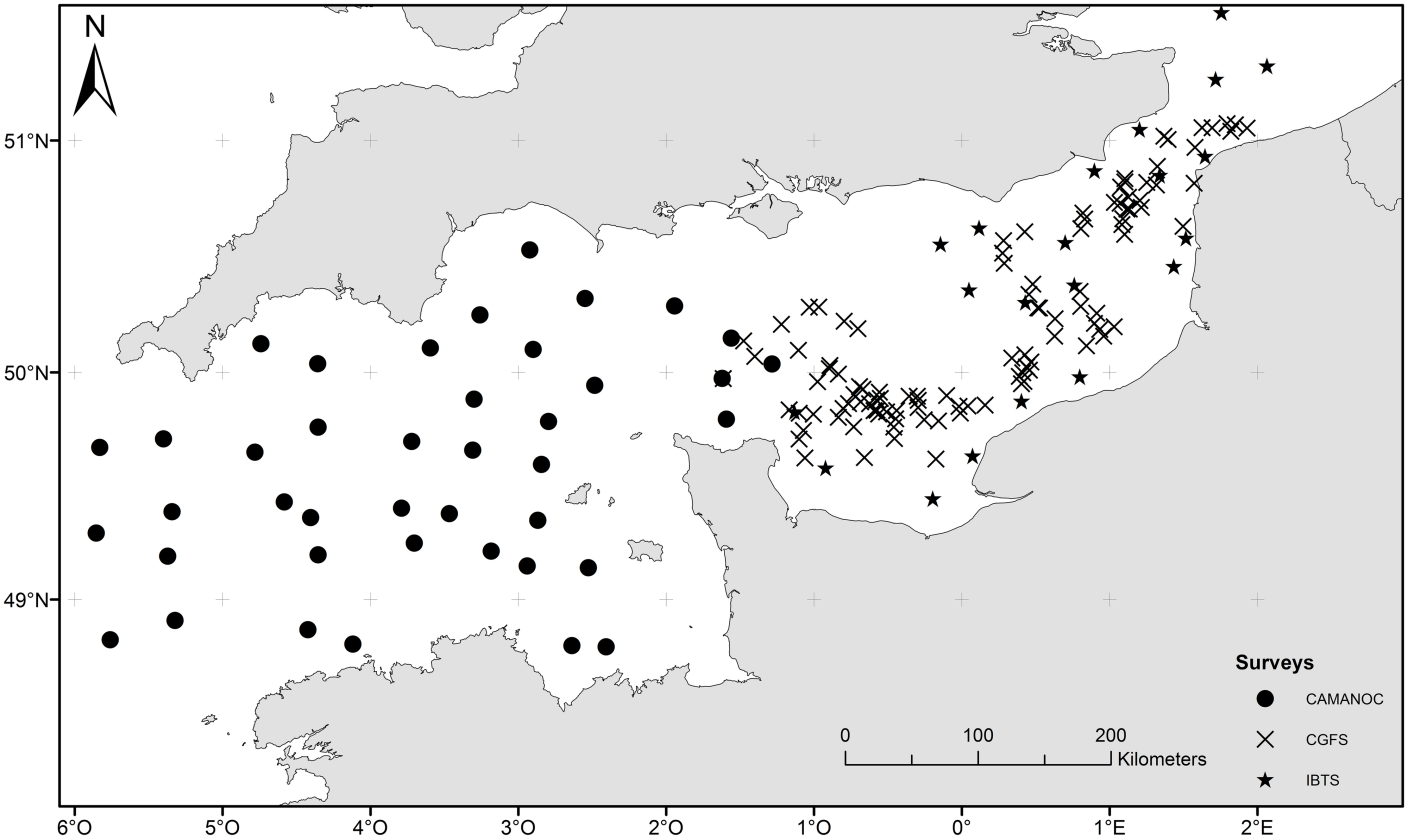
Station locations for the three scientific surveys carried in the study area.

IFREMER contributes to the collection of basic biological data in the Eastern English Channel and North Sea through its annual bottom trawl surveys, the CGFS (Channel Ground Fish Survey, carried out in October since 1988 on board of the RV *Gwen Drez* [56]*)* and the IBTS (International Bottom Trawl Survey, undertaken in January/February since 1970 on board of the RV *Thalassa* [57]). Since 2006, all megabenthic invertebrates captured in the trawl have been identified, counted and weighted. Additionally, in September 2014, IFREMER carried out a bottom trawl scientific survey, CAMANOC (Campagne Manche Occidentale [58]), on board of the RV *Thalassa* in the western English Channel, where megabenthic invertebrates caught in the trawl were also identified. This set of surveys all had a random stratified sampling strategy but with varying intensity depending on the covered survey area. Their data merged together cover the entire study area although there are much more observations and longer time series in the eastern part of the Channel than in the western part (Table 1 and Fig 2).

**Table 1:** Number of *in situ* observations used in this study.

	SURVEY (number of trawls)		
Year	CAMANOC	CGFS	IBTS
2007		18	
2008		91	11
2009		87	14
2010		97	24
2011		97	12
2012		84	20
2013		84	20
2014	35		17

For all three surveys, the sampling gear used was a Very High Vertical Opening (VHVO) bottom trawl (or “GOV”), well adapted for catching demersal species (in particular fish and cephalopods), with a 10 mm mesh size at the cod-end for catching juveniles. The sampling strategy was using randomly stratified and standard 30 minutes hauls at 4 knot speed, evenly distributed over the whole study area. Demersal species and megafauna/epifauna caught in the bottom trawl were sorted, identified, counted and weighed. Although bottom trawl is seldom recognised as a valid sampling device for benthic invertebrate species, such observations are nonetheless believed to be particularly relevant as 1) they represent the benthic fauna fraction the most likely affected by bottom fishing and 2) they integrate assemblages’ composition over large areas (3–4 km) and are more representative of larger scale habitat structure. Bottom trawling mostly targets fish and cephalopods while mega-zooplancton and most other benthic invertebrates are considered a by-catch of this fishing technique and are discarded at sea. The present analysis focused on species assemblages limited to benthic invertebrates other than cephalopods, the composition of which adequately illustrates both habitats and impact gradients [59]. Species or taxonomical group biomass was chosen as indicator of the presence of colonial and encrusting species that cannot be quantified. Log-transformed biomasses were expressed as a proportion of the total benthic megafauna/epifauna biomass of each trawl to account for difference of capturability of benthic organisms depending on different seafloor and bottom gear types.

Moreover, the sampling was centred on the same period of time (autumn-winter), when the benthic communities are stable. Furthermore, some works proved that globally benthic communities are mostly stable over time at the scale of the English Channel [60], [61], [62], [63].

No permissions were required as this study is based on already-existing datasets. *In situ* data did not involve endangered or protected species.

The species nomenclature used was standardized using WoRMS database. In order to avoid bias in the identification of fauna, some taxa were regrouped in higher levels, with minimal loss of information on the community structure [64], [65], [66]. The following procedure was used: all taxa expressed at the species level were first aggregated at the genus level. Then to be kept at that taxonomic level, a given genus had to be observed over at least 6 different years (consistently recorded over the 8 years’ time series) otherwise it was iteratively regrouped into a higher taxonomic level (family, order, class, division) following the same criteria. If a given division group was observed in less than 6 different years, it was removed from the analyses. This resulted in removal of 48 taxa and into the aggregation of the 394 remaining taxa into 120 taxonomic groups (S1 Table).

### Biological traits

Biological traits of species were defined using the BIOTIC database [67] and from information given by Garcia [68], Le Pape *et al*. [69] and Brindamour *et al*. [70]. For missing biological traits, additional information was obtained from published literature. Taxa for which necessary information was not available were excluded from further analysis.

The five functional traits (Position, Feeding, Motility, Size, Fragility) were selected based on the knowledge of the response of benthic taxa to trawling disturbance (Table 2) [31]. They reflect respectively the ability to avoid direct gear impact, to benefit from trawling for feeding, to escape gear, likelihood to get caught by the net and ability to resist trawling/dredging action. Each of these characteristics is either advantageous to survival or demonstrates sensitivity to trawling. To allow quantitative analysis, a score was assigned to each category, varying from low vulnerability (0) to high vulnerability (3). Scores across all five categories were then summed for each taxon (the highly vulnerable taxon could reach the maximum score of 15) and this value was considered as a species-specific index (SI) of sensitivity to trawling disturbance [71].

**Table 2:** Five categories of biological traits and their respective scoring scheme (from [31]).

Sensitivity scores	Position	Feeding	Motility	Size	Fragility
0	Deep burrowing	Scavengers	Highly mobile (swimming)	Small < 5 cm	Hard shell, burrow, vermiform, regeneration
1	Surface burrowing (first cm)	Deposit feeders/predators	Mobile (crawling)		Flexible
2	Surface		Sedentary	Medium 5–10 cm	No protection
3	Emergent	Filter feeders	Sessile (attached)	Large > 10 cm	Fragile shell/structure

When taxonomic groupings, at higher taxonomic level (such as family, order, class or division) were considered, the homogeneity of SI within each group was assessed by following means: if the standard deviation (SD) of any given indicator score (*e*.*g*. position, feeding and so on) for a group was above 1.5, and if the SD of the SI for the species in the group was above 2.5, the group was removed from the analysis. For some group, this could result in their removal, such as Bivalvia group which was found to be too heterogeneous as it accounted for 22 different initial taxa with very different functional traits (S1 Table), or their conservation, such as Polychaeta for which we maintained species well catched by the gear. The maximum value of the regrouped species indicators was chosen to avoid over-looking particular species sensitivity traits. Finally, a weighted average of each taxon indicators was computed using their relative biomass data for each trawl observation, then summed to obtain the trawl disturbance indicator, TDI). Thus, this TDI incorporates organisms that are mostly considered as by-catch, since the type of bottom trawl used is mostly targeting demersal fish and cephalopods. Some of those organisms are highly vulnerable to trawling, *e*.*g*. sponges, bryozoans, large ascidians or hydroids (higher TDI values) while others have low vulnerability (lower TDI values; they could even benefit from trawling disturbances and exhibit high survival rates when discarded), *e*.*g*. sea stars, hermit and swimming crabs.

TDI is calculated as:

$$TDI_{x}=\;\overset{N_{x}}{\underset{1}{\sum }}\frac{Bi_{x}}{Bn_{x}}\;\times \;SI_{i}$$

with *TDI*_*x*_, TDI of the x^th^ observation; *N*_*x*_, the number of taxons in the x^th^ observation; *Bi*_*x*_, biomass of the i^th^ taxon in the x^th^ observation; *Bn*_*x*_, total biomass of the x^th^ observation and *SI*_*i*_, SI of the i^th^ taxon.

### Fishing pressure

**Fig 3:**
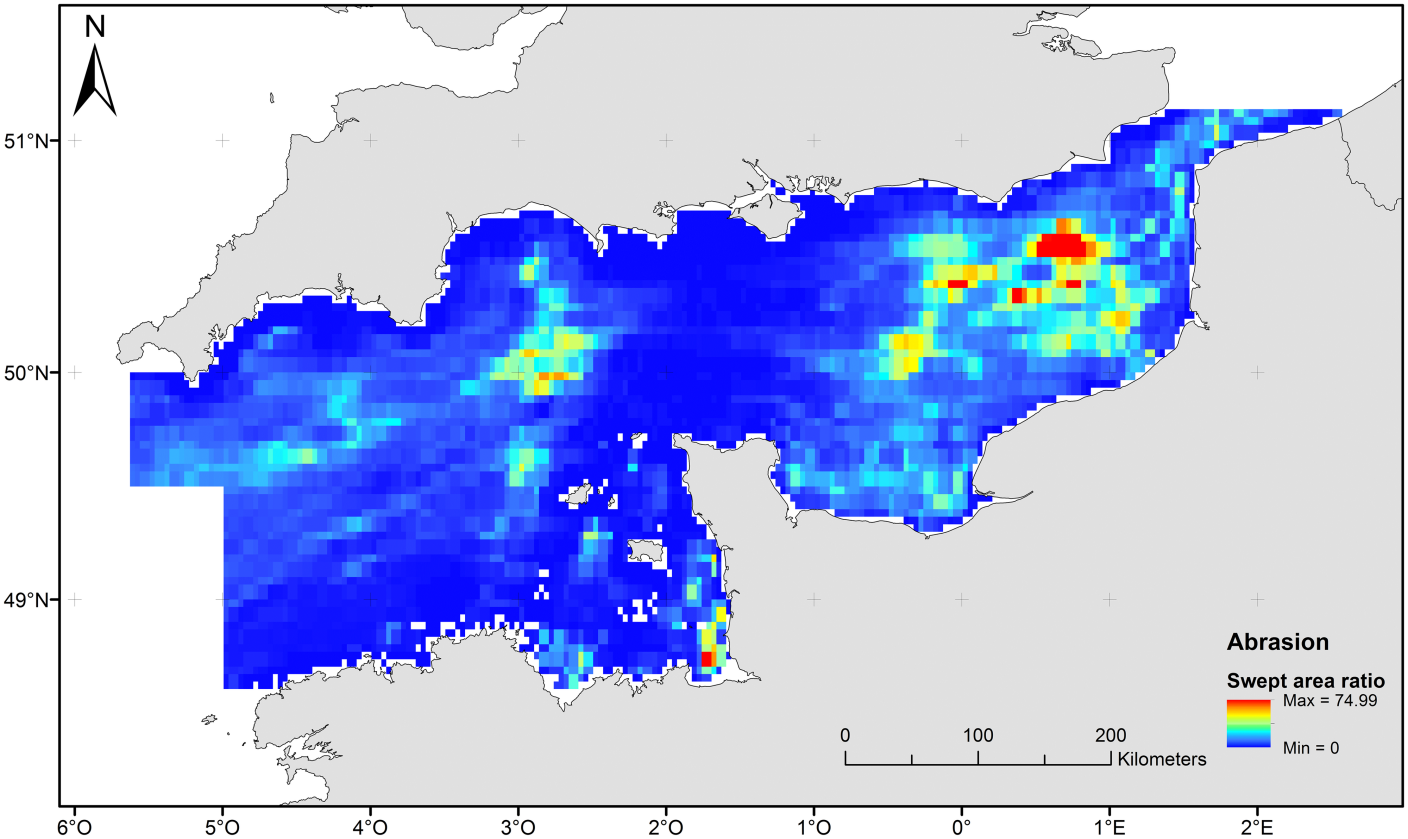
Trawling abrasion estimation for the English Channel.

The distribution of impact of fishing on the seafloor was estimated from aggregated fishing effort data in 3x3’ resolution, expressed as total hours fished. Original data were obtained from international Vessel Monitoring System for ground-towed gears (beam trawlers, dredgers and otter trawlers). This work was undertaken by the ICES Working Group of Spatial Fishery Data following OSPAR request on mapping of bottom fishing intensity using VMS data [72], [73]. The area of abrasion of the seabed was computed from vessel speed, fishing duration and gear type and expressed as the percentage of the total area of each 3x3’ cells. A spatial layer of the maximum percentage of bottom surface sustaining superficial abrasion by trawling was computed over the 2009 to 2013 period (Fig 3). Fishing pressure will be therefore referred as abrasion from this point onward.

### Statistical analyses

*In situ* data (both species composition and TDI were coupled to the predicted layers resulting from the PDS approach (SfG, Dist and Risk) and to the fishing pressure (abrasion) layers based on geographical location.

### BIO-Env procedure

The BIOENV analysis [74] compares the distance/similarity matrices between two sets of data having either samples or variables in common, and informs on environmental variables that best correlate to sample similarities of the biological community. The BIO-ENV analysis has already been used in previous studies [75], [76], [77] to relate community structure to environmental predictors. We compared similarity matrices of different combination of 13 environmental parameters, including initial variables (i.e. depth, waves, bottom currents, sediments, mean temperature, interannual and intraannual temperature variability, mean salinity and food availability), modelled descriptors (i.e. disturbance, scope for growth and risk) and abrasion, to a similarity matrix of the community based on relative species biomass. Spearman rank correlation coefficient was calculated between the two matrices (environmental similarity and community similarity). Thus, the best combinations of environmental variables was identified (ordered by Spearman correlation coefficient values) and subjected to a permutation test to determine its statistical significance. The tests were performed using PRIMER 6 software [78].

### Principal components analysis (PCA) and Redundancy analysis (RDA)

PCA was undertaken to explore and illustrate the relationships between the nine environmental descriptors used to construct the PDS indices and the PDS indices (disturbance, scope for growth and risk) themselves and abrasion. This PCA was computed on the correlation matrix between these nine variables. The same approach was undertaken to explore the relationship between PDS indices, TDI and abrasion.

PCA can also be seen as an indirect gradient analysis that employs a linear response model to produce a simple approximation of the species response along an environmental gradient [79]. It was performed on the Hellinger distance matrix of the relative biomass composition data [80], [81], [82]. PDS (disturbance, scope for growth and risk), TDI and abrasion were represented passively on the biplot as supplementary variables. Redundancy analysis (RDA) is a direct extension of multiple regressions to modelling multivariate response data [82]. It is a constrained version of PCA in that the ordination axes are linear combinations of the explanatory variables. This method was used in combination with Monte-Carlo permutation tests to explore the multi-linear relationships between benthic community and PDS indices. In the Monte-Carlo permutation test, the reference distribution is simulated by repeatedly permuting the samples. A statistical test (F-ratio) is computed for the original data and compared with those of permuted data. The value of the significance test is the probability that the response is independent from the tested explanatory variable. Variance partitioning was used to determine the proportion of variation attributable to or shared among different variables [81], [83].

### Generalised Linear Model (GLM)

The relationship between the different modelled indicators was explored by means of Spearman correlation coefficient tests. This non-parametric test is based on ranks and does not assume any particular shape in the relationship. Visual exploration of the shape of the relationships did not lead us to consider that non-linear models would make much interpretable difference and a linear approach was preferred. A Generalised Linear Model (GLM, [84]) linking the TDI as a response to both PDS indices was first developed. TDI value was log-transformed to improve its distribution and a Gaussian link function was chosen. A second model adding abrasion resulting from seabed impacting fishing effort to the PDS indices was also developed. This later model was then used to produce a predicted TDI map, covering the entire studied area using both PDS indices maps and the abrasion map as predictors.

These multivariate and univariate analyses were implemented in R (R-3.2.1, 2015) using the vegan [85] and MASS [86] packages.

## Results

### Process-driven sensitivity (PDS)

#### Disturbance

**Fig 4:**
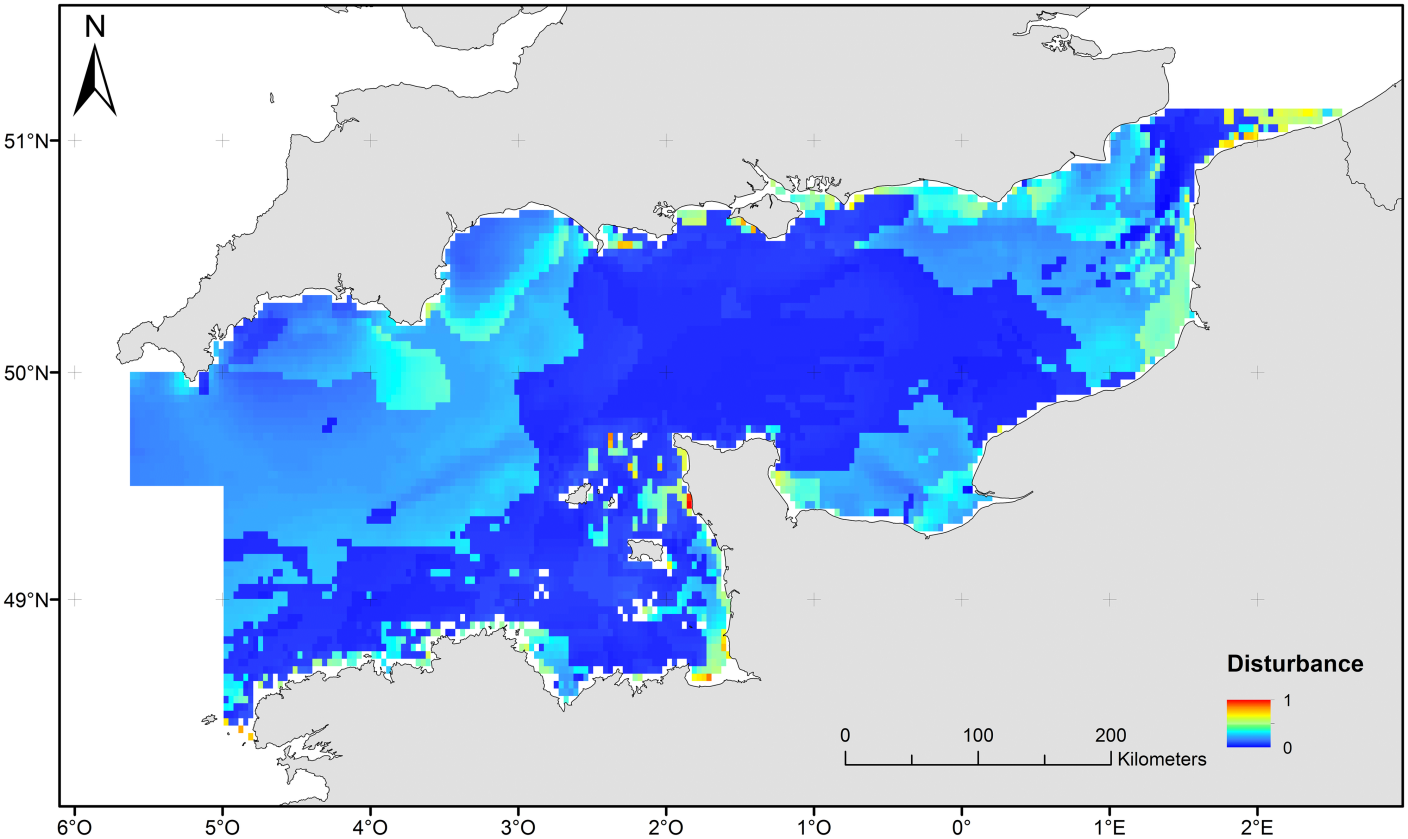
Map of disturbance index for the English Channel.

The map of disturbance index showed areas with relatively stable surficial sediments as well as areas which are relatively more disturbed (Fig 4) [87].

Naturally disturbed areas (in yellowish and reddish colors in the map) correspond to sandbanks located in the southern North Sea or near the Dover Strait where even relatively weak currents can easily re-suspend fine sediment particles. Areas characterised as stable (in blueish colours) correspond to coarser, gravel dominated sediments, which are difficult to re-suspend even when strong current friction occurs. Because of this interaction between sediment grain size and current, in the study region, the habitats characterised as low disturbance (Fig 4) correspond to areas with coarse grained sediments and high tidal current friction.

#### Scope for growth

**Fig 5:**
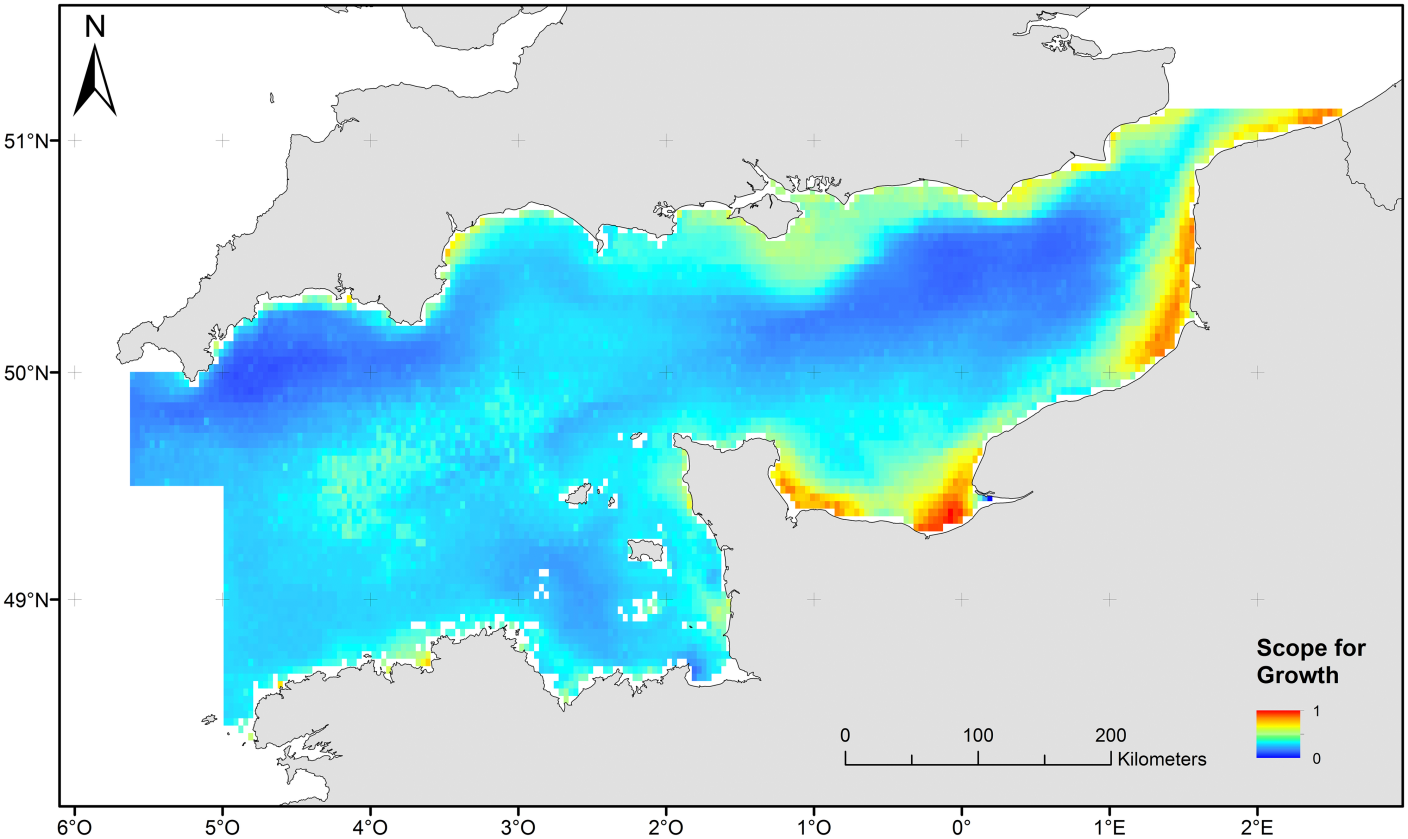
Map of Scope for growth of the English Channel.

Areas with high scope for growth (red colours) are located near the coasts where food availability and, to a lesser extent, temperature-derived variables, are the most favourable (as assumed in our model) for benthic organisms (Fig 5) [87]. The role of food availability is highlighted by strong SfG values (Fig 5) observed near estuaries and river plumes.

#### Risk of habitat damage

**Fig 6:**
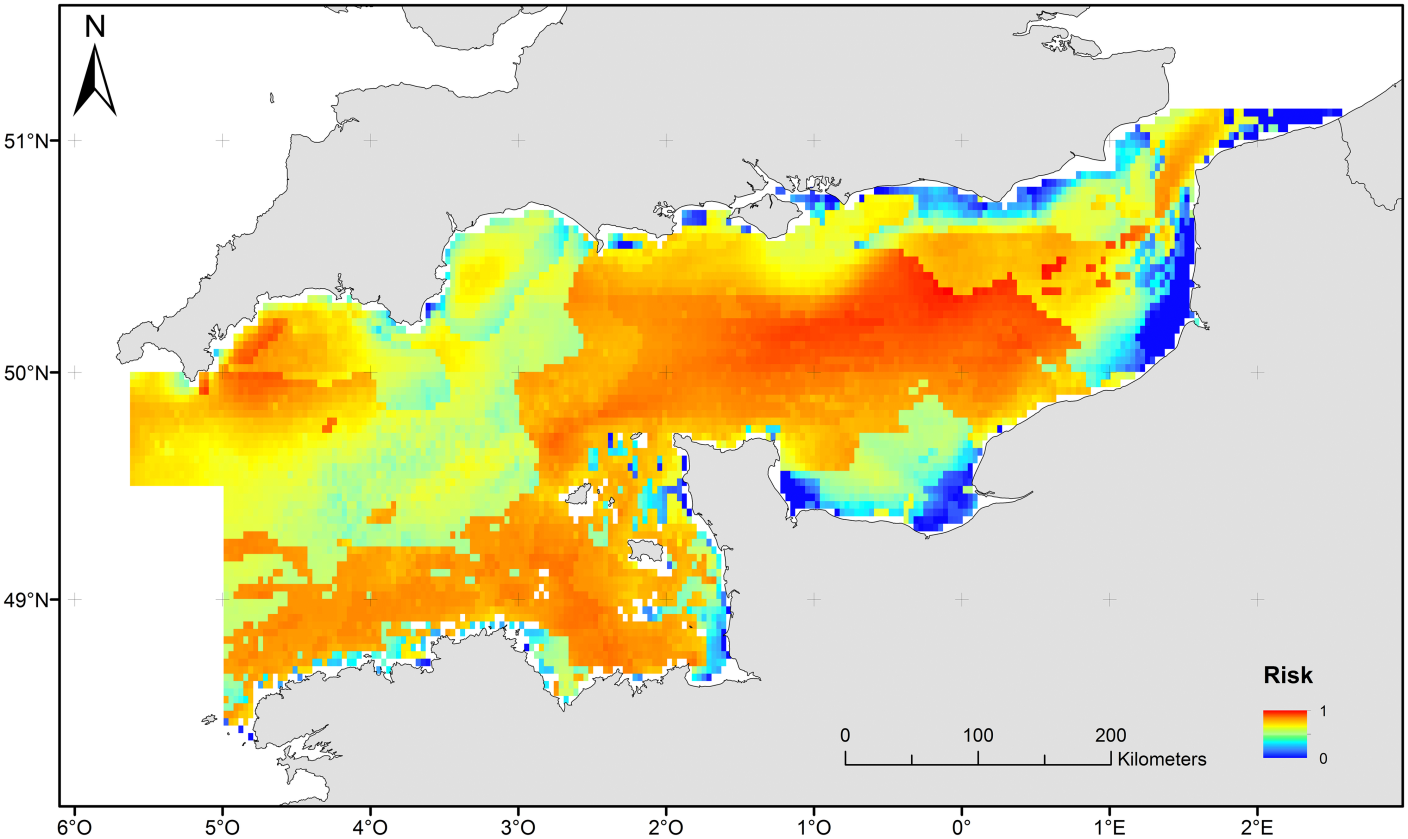
Map of risk/ sensitivity of benthic habitats and communities to trawling damage.

The risk map (Fig 6) [87] combined both processes described above and is interpreted as a process-driven sensitivity of benthic habitats and associated communities to trawling damage. Low risk (blue colours) was found in the bay of Seine (Fig 1), as well as sandbanks and coastal areas. The major part of the English Channel is covered by habitats that can be considered from medium to high risk (Fig 6, from orange to red colours).

#### Relationship between initial descriptors and PDS

**Fig 7:**
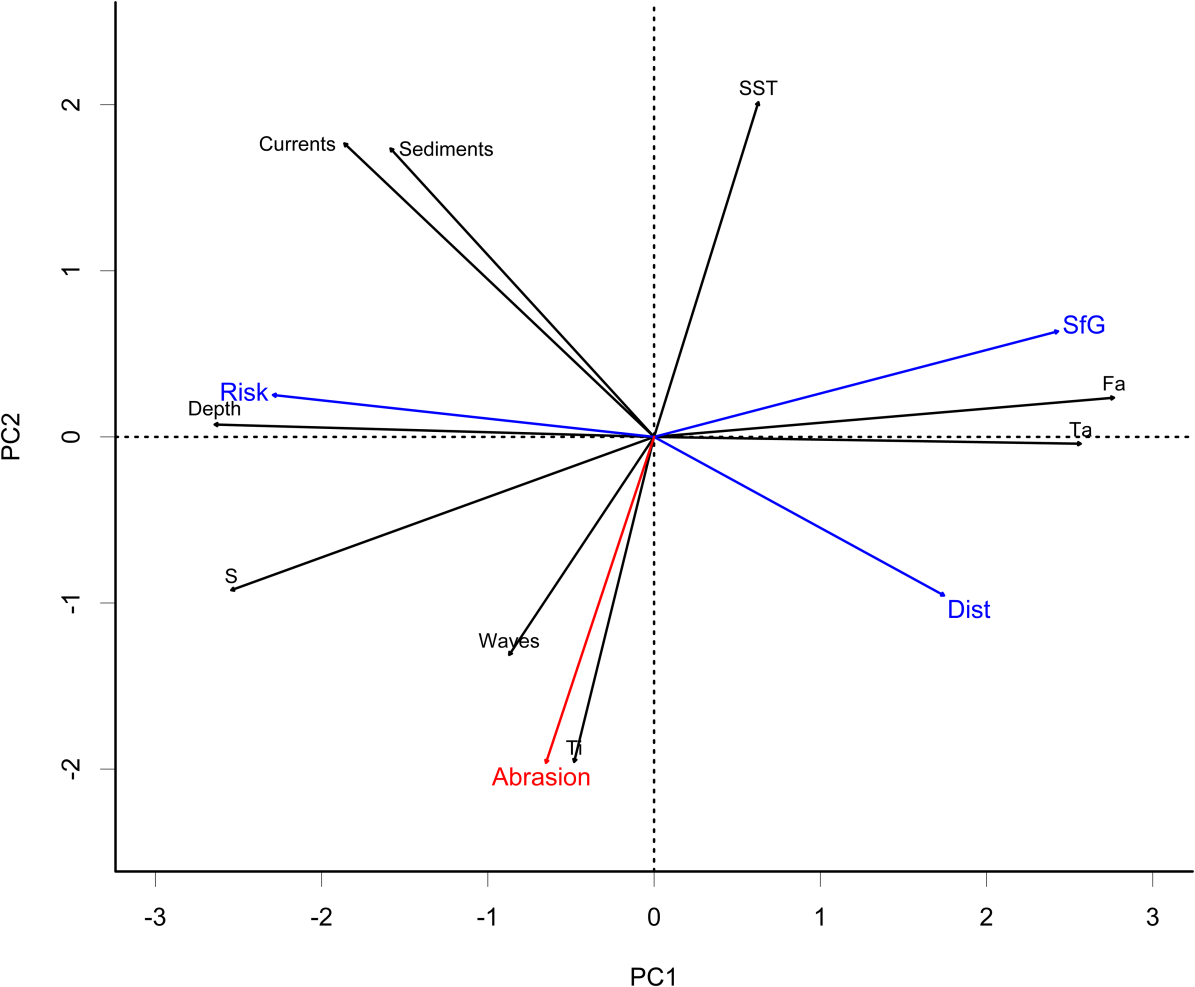
Results of PCA analysis for PDS predictors and indices. Disturbance, Scope for Growth, Risk and seabed abrasion are shown as supplementary variables.

Nine environmental predictors were used to construct the PDS: depth, waves, bottom currents (currents), sediments (grain size), mean temperature (SST), inter-annual temperature variability (Ti), intra-annual temperature variability (Ta), mean salinity (S), food availability (Fa), which resulted in modelling three PDS indicators: disturbance (Dist), scope for growth (SfG) and risk. The relationships between the different predictors used to produce the PDS and the PDS indicators themselves were illustrated using a PCA (Fig 7). The first two PCA axes represented respectively 62.5% and 19.7% of the variance. Disturbance, Scope for Growth, Risk and abrasion from bottom impacting fisheries were added as supplementary variables (blue and red arrows).

In the present study, we found that scope for growth is mostly related to food availability and intra-annual temperature variability as well as negatively correlated to depth while disturbance has negative relationship with bottom currents and grain size. Risk was negatively correlated to disturbance and scope for growth, as per definition (Fig 7). Seabed abrasion, being an anthropogenic impact, did not seem to be related to any of the PDS indices.

### Community structure and PDS, TDI and fishing abrasion

#### BIO-Env procedure

The 13 variables used in the analysis (Fig 7) were tested in a BIO-ENV procedure to evaluate their relation to benthic community structure. Among the environmental variables considered, the combination of bottom currents, waves, intra-annual temperature variability (Ta), disturbance (Dist) and scope for growth (SfG) were identified as best explaining species composition (ρ = 0.384), explaining 14.8% of community variability (S2 Table). In this analysis, bottom currents, intra-annual temperature variability and Disturbance were always selected by the procedure. Selection frequency varied for the other variables: scope for growth (62%), waves (61.5%), inter-annual temperature (Ti, 30.8%), depth, sediments and salinity (23.1%). Risk, abrasion and food availability were never selected. This correlation decreased when environmental variables were considered individually. When considered individually (all Bio-Env values not presented here), the most important variables were, in order of decreasing correlation, depth (ρ = 0.340), intra-annual temperature (ρ = 0.311), food availability (ρ = 0.279), bottom currents (ρ = 0.268) and risk (ρ = 0.220). The individual correlation values for SfG and Disturbance are respectively ρ = 0.217 and ρ = 0.173. The correlation of community similarity with the combination of SfG and Dist was ρ = 0.239, meaning that it explains 5.7% of variability. The strong relationship between disturbance and bottom currents suggested that these variables were probably equivalent in term of explanatory power of the community structure. Similarly scope for growth was positively correlated to intra-annual temperature and food availability and negatively related to depth. The fact these came out as the most relevant predictors of the benthic community structure confirmed the predictive power of the PDS indices.

**Fig 8:**
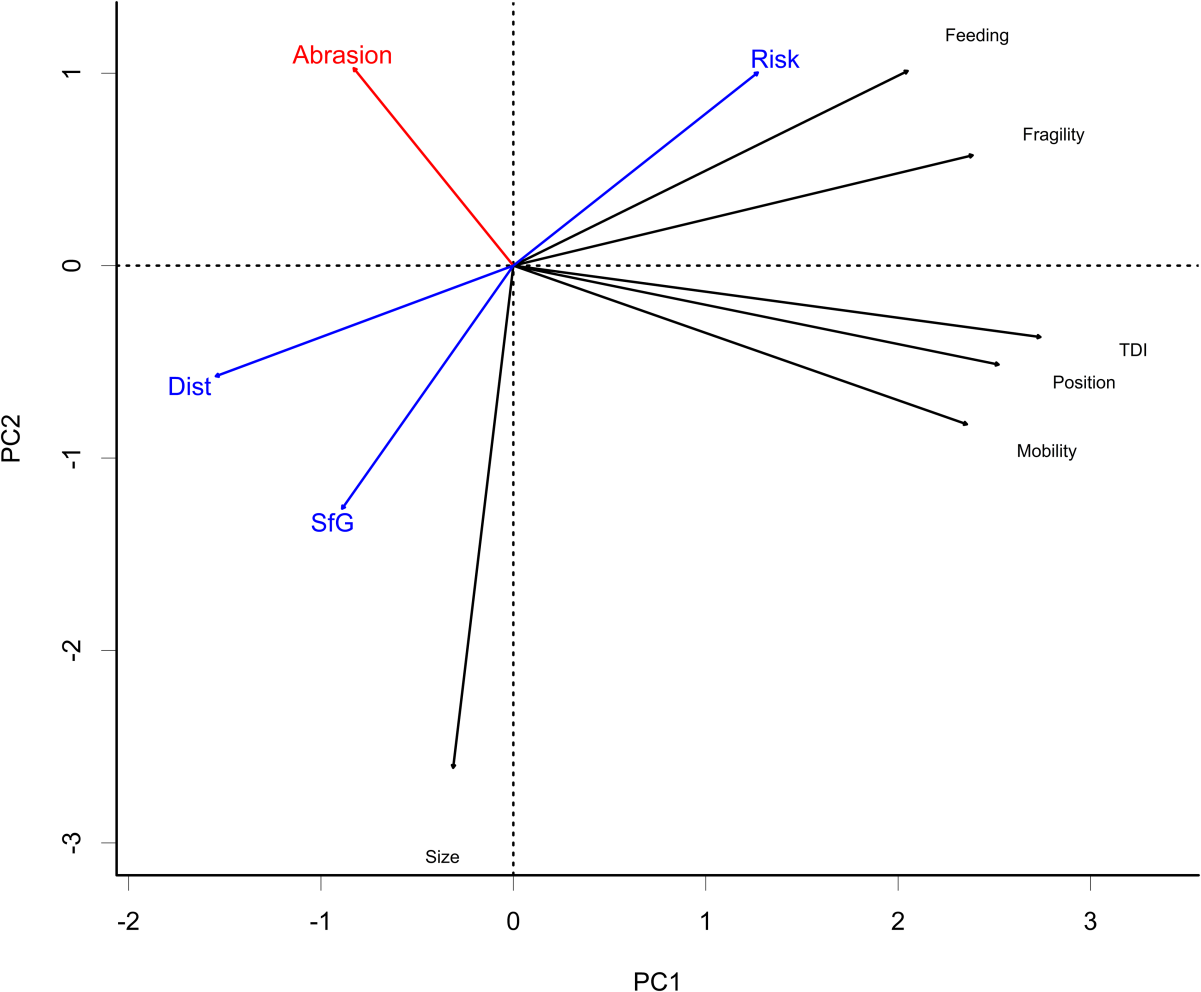
Results of PCA analysis of functional traits vs PDS indices, TDI and abrasion.

The relationship between the three PDS indicators and functional sensitivity traits was analysed using PCA (Fig 8) and Spearman correlation tests (S4 Table). Sensitivity, mobility, position and fragility showed some significant positive correlation with each other while their relations to size were much weaker if any. The first two axes represented respectively 44% and 21% of the variation.

Scope for growth and disturbance were significantly correlated (ρ = 0.554) to each other and, by design, negatively correlated to risk (ρ <-0.8). Mobility, position, fragility and feeding were positively and significantly correlated to each other and their variation was reflected in the overall TDI. Size was less correlated to other indices with positive and negative relationship depending on the index considered (Table 3). Risk and TDI were found to be significantly correlated (ρ = 0.237). Abrasion was found to be significantly correlated (ρ = 0.239) to risk and significantly negatively correlated to TDI (ρ = -0.261). Visual exploration of the shape of the relationships did not lead us to consider that non-linear models (such as CCA or GAM) would make much interpretable difference.

**Table 3:** Spearman correlation matrix between the functional sensitivity traits and the other factors.

	Mobility	Fragility	Position	Size	Feeding	TDI
Depth	0.001	- 0.005	-0.016	-0.019	0.030	0.057
Waves	0.081	0.039	0.037	0.058	-0.004	0.057
Currents	-0.017	0.085*	0.006	-0.089*	0.064	0.041
Sediments	0.003	0.058	0.019	-0.023	0.045	0.037
SST	-0.058	-0.040	0.006	-0.020	-0.016	-0.078*
Ta	0.060	-0.021	0.053	0.100	-0.003	0.049
Ti	0.037	0.017	0.043	-0.003**	-0.024	-0.002
S	-0.016	-0.005	-0.039	0.003	0.011	0.017
Fa	0.019	0.014	0.040	-0.012	-0.016	-0.008
Dist	0.013	-0.040	0.007	0.007	-0.027	-0.020
SfG	-0.003	0.007	0.003	-0.045	-0.025	-0.032
Risk	-0.011	0.020	-0.013	0.009	0.033	0.027
Abrasion	0.068	-0.015	0.063	0.087*	0.012	0.037

**p<0.01,

*p<0.05

**Fig 9:**
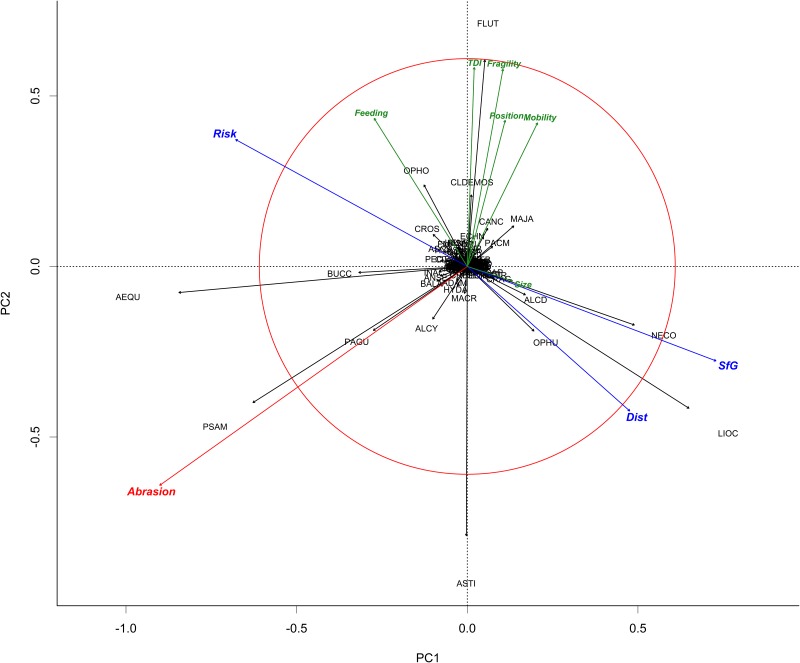
Results of PCA analysis of the taxonomic composition data with fragility, feeding, mobility, size, position. Disturbance (Dist), scope for growth (SfG), risk, TDI and abrasion are represented as supplementary variables.

The relationship between genera of benthic mega-epifauna and PDS indicators was analysed using PCA (Fig 9). The first two axes represented respectively 14% and 11% of the variation, highlighting the lack of strong gradient and structure in observed community. It is therefore expected that a large part of the variation in the community structure will remain unexplained and should be considered as white noise at the spatial scale of the study.

Relative biomass of *Necora*, *Liocarcinus*, *Ophiura* and *Asterias* was correlated with high SfG and natural disturbance (*i*.*e*. low risk). At the opposite, genus *Aequipecten* is linked to high risk areas (Fig 6). Genus *Psammechinus* is associated with high fishing effort impacting seafloor. Genus *Flustra*, *Ophiotrix*, *Maja*, *Cancer* and Demosponge species were associated with higher TDI (all functional traits but size). They were not correlated to variables selected to define disturbance or scope for growth and weakly negatively correlated to abrasion. Finally, genus *Asterias* is strongly dominant and negatively correlated to TDI (all functional traits but size)

The variance partitioning and Monte-Carlo permutation procedures showed that disturbance and scope for growth axis explain significantly 5% and 8% of the community structure variance respectively, out of which 3% are shared between the both. Interestingly, fishery driven abrasion pressure explained about 2% of the variance illustrating its relatively small but still significant correlation with the community structure.

A GLM model was built to link the TDI to risk and abrasion (S3 Table). Both explanatory variables were found statistically significant (p < 0.001) and the addition of abrasion into this model increased its predictive ability. The Spearman correlation between the measured TDI and TDI predicted by the GLM model rose from 0.24 to 0.38 when the abrasion was used.

**Fig 10:**
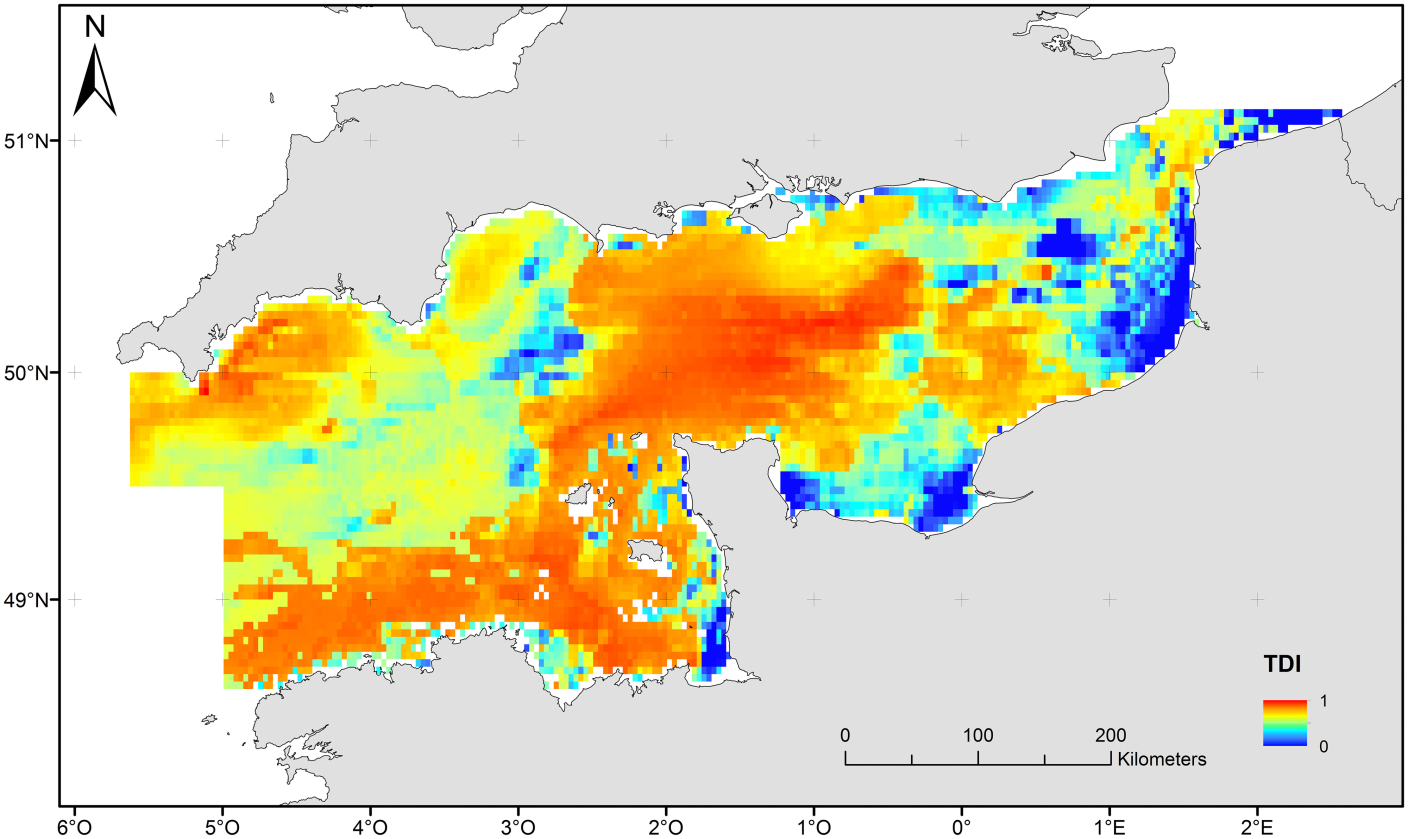
Map of estimated TDI predicted from the risk and abrasion distribution.

Regression coefficients of this model were used to produce a map of estimated TDI from the risk and abrasion distribution maps (Fig 10).

Highest values of estimated TDI are located in the coarser sediment areas and the overall spatial pattern appeared to be a contraction of the area of high risk identified in the initial PDS map (Fig 6).

## Discussion

A considerable rise in seabed habitat mapping has followed the recognised need for accurate spatial benthic marine environmental data to inform an ecosystem-based approach to ocean management [88]. The application of the modified approach developed by Kostylev and Hannah [14] to the English Channel habitats seems pertinent as this conceptual model summarizes environmental parameters that could be easily related to biological traits. The correct implementation of this approach requires modelling of two descriptors: the scope for growth and the natural disturbance.

### Relationship between PDS indicators and the Channel environment

The developed habitat model reflects the main ecological characteristics of the benthic habitats expected to arise from the effects of natural processes on biological traits of benthic species. Habitats with low natural disturbance are areas with low reworking of the surficial sediment by natural processes, allowing the establishment of a rich sessile epifauna community, with K-strategy species being common. Habitats with low SfG impose a cost for species to grow and reproduce because of shorter supply of food and higher energy expenses for adaptation to environmental stresses. In habitats combining low disturbance and low SfG, large suspension-feeder species with long life span and slow growth can often be found [14], [89]: these species are more vulnerable to added (anthropogenic) disturbance.

### Process-driven sensitivity in the English Channel

Coarse surficial sediments are dominant in the English Channel, such as pebble sized gravel [39], [46]. The whole English Channel is characterized by a relatively low level of natural seabed disturbance, which may favour the development of benthic biomass [14]. Areas where disturbance is high have unstable substrates because of finer sediment grain size and higher hydrodynamic energy. They mainly correspond to the fine sediment areas in the southern bight of the North Sea and coastal areas near the Dover strait and the south-western coasts of England. In these shallow areas, storms cause active sediment resuspension due to wave action [90], [91]. At the same time, areas characterized by high scope for growth are located along the coasts mostly due to high food availability and favourable water temperatures. Other areas may therefore be associated with potentially slower recovery of benthic communities from bottom disturbances and biomass removals [14].

### Environmental processes modelled

Our results (*e*.*g*. Bio-Env) have shown that Disturbance and Scope for Growth significantly explain spatial variability in benthic community by integrating most relevant environmental variables (such as waves, currents, temperature, sediments and salinity).

In other studies, the importance of individually selected environmental variables depended on the considered location, and on their ability to limit or influence character of species assemblages. For example, in the Baltic Sea, salinity is the factor significantly responsible of the benthic community distribution [92]. On the German Bank (Scotian shelf), Todd and Kostylev [77] reported that the single variable that best explained the distribution of bottom fauna was the summer oxygen saturation level. In our study, the oxygen saturation is nearly constant all the year, due to shallow depths and mixed waters masses, as was also observed by Borja et al. [93] and Galparsoro *et al*. [24] on Spanish coasts. The English Channel, located in a temperate region with no extreme hydrological variations through the year, can be considered as a moderately stable area. As a consequence, wave action, tidal currents and the resulting sediment dynamics become the main factors influencing benthic assemblages. The effects of wave- and tide-generated currents are enhanced by the overall shallow depth (<150 m), as observed in other studies [24], [94]. These environmental variables are integrated in the calculation of disturbance (waves, currents and sediment) and scope for growth (depth) axes.

### Ecological interpretation of PDS

PDS map may help predict which biological traits will be most likely to occur depending on the site environmental characteristics. Most of the English Channel exhibits low to medium scope for growth levels and low natural disturbance levels. Organisms living in a low disturbance environment with low scope for growth are potentially long-lived, slow-growing, slow reproducing and have limited dispersal [14]. These species are likely to be more vulnerable to human activities, such as trawling, dredging, mineral extraction or direct fishing. In contrast, in this study, coastal areas were often identified as highly disturbed and characterised by high scope for growth, resulting in communities favouring either adversity tolerant species or species defined as *r* strategists (opportunistic species) [95]. According O’Boyle *et al*. [96], it would be convenient if scope for growth was associated to community recoverability and disturbance—to habitat vulnerability. However, some elements involved in modelling of sensitivity (risk) may be relevant to both SfG and Disturbance and thus a one-to-one correspondence may not always be valid. Notwithstanding this, low scope for growth generally infers low community or species recoverability from impact while high habitat stability is associated to organisms’ relative vulnerability to physical disturbance. Thus, highly sensitive benthic communities would likely be found in stable, adverse environments while benthic communities with low sensitivity should be found in disturbed, benign environments [14]. The areas with low Scope for Growth and high Disturbance will support communities with species tolerant to adversity. The areas with high Scope for Growth and low Disturbance will support communities with mixed r/K strategies species. We assume that moderately sensitive communities would be found in the other areas of the English Channel, the characteristics of which depend upon the relative degree of influence of scope for growth and disturbance. Because of the approach, we characterised sensitivity on a relative, not absolute, scale; the terms high/low/moderate apply within the constraints of the studied areas, with reference to end points of characterisation.

### PDS link to taxonomic composition and TDI

Spatial heterogeneity at scales finer than the map resolution is often perceived as a limitation to the development of habitat maps [97]. Most published studies on benthic communities are very detailed and focus on small-scale patterns of diversity, distribution and structure of coastal biota [24], [98]. Information on offshore benthic species and assemblages at large-scale is required for spatial planning of conservation and human activities or for integrated assessment such as MSFD. The use of scientific surveys, aiming at monitoring fish stock, enables gaining this knowledge from the observation of macro-epifauna and megafauna over wide geographical areas (see [59] for review). Moreover, the impact of fishing on infauna, which is typically used for quantitative benthic studies [99], was shown to be undetectabla in some habitats [100], further supporting the use of bottom trawl observations for monitoring benthic sensitivity [89]. However, the integration of observations over large areas, as in the case of bottom trawling, may also generate certain randomness in the observations of usually over-dispersed organisms. This would result in a large amount of unexplained variance in the data as was found in this study. Still, the use of bottom trawls samples, integrating mega-epifauna over large distances (about 4 km in average), was proven useful here to address mesoscale variability and describe bio-regional patterns. At the same time, there is a concern that certain biological traits are underrepresented in the trawl samples–*e*.*g*. encrusting species and deep burrowing infauna. However, this fact only interferes with our interpretation of the influence of environmental factors on community structure, while the interpretation of TDI is unaffected, as the trawling samples by their very nature recover the organisms the most affected by trawling. The use of TDI to identify the most sensitive taxa and measure the observed *in situ* sensitivity was particularly useful. It highlighted areas where community sensitivity to additional impacts may be important (see [101], [102], [103] for examples). It is notable that there is a significant correlation between the assessed risk to seabed communities and habitats and estimated TDI values. This justifies the use of habitat template-based PDS approach to characterisation of benthic habitats and confirms that it portrays habitat and community sensitivity properly. Because we calculated “Risk” as the distance from the disturbed productive environment along habitat template axes, it implicitly incorporates assumption on change of life history and biological traits of benthic animals. Correlation to TDI values proves that indeed our operational characterisation of environment captured assumed related changes in biological traits. In particular, it showed that it is species which are most likely to be affected by trawling (large fragile sessile filter feeders) which are prevalent in ‘high risk’ areas. The PDS predictors were also significantly correlated to the community composition and species associated with areas of higher disturbance (*e*.*g*. *Liocarcinus*, *Pagurus* or *Asterias*), where the re-establishment and the survival of the organisms could be affected [104]. Mobile predators and scavengers could be attracted to such areas by potential prey [105] damaged by trawling and thus benefit in these feeding areas from trawling activities [106], [107].

Some other species, such as sponges, are linked to high sensitivity areas (low Disturbance, low SfG) because these medium-large size, long life span, suspension feeding and low mobility species need more stable environments to live. Recovery of these species may take a long time in case of large-scale disturbance [108].

Note that the first two PCA axes represented 14% and 11% of the variation in community structure respectively, and Bio-Env analysis showed that about 15% of the community structure variability is explained by environmental factors. These values seem low, but the objective of the Bio-Env method is in defining suites of variables which ‘best explain’ the biotic structure, and actual values depend on taxonomic resolution and accuracy of the environmental descriptors used. In the top ranked combinations, Disturbance was always selected, followed by SfG (selected 62% of time) and by waves climatology (61.5% of time), suggesting that our estimation of Disturbance and Scope for Growth was meaningful.

### Limitations of the PDS

The assumption of ‘natural environment’ is a common limitation in habitat mapping in general, as temporal dynamics, climate change, natural ecosystem dynamics and human activities all affect the types and abundances of organisms living in a region at a particular time. Seafloor impacts of fishing (by trawling or dredging) was shown to be the most important human pressure in terms of its spatial extent and level of impact [109], [110], [111], [112]. Bottom trawling gear produce scours on the seabed, the depth and width of which depends both on sediment and gear type [113], [114], [115], [116], [117]. The use of bottom trawling gear is associated with detrimental impacts on the marine benthos [114], [118], [119], [120], [121] and biomass, abundance and cover of macro-fauna and -flora on soft and coarse sediments have been shown to be negatively correlated with trawling intensity [122]. Focusing on the impact of fishing on the seafloor has particular implication on the ecosystem functioning as upright sessile epifauna (scoring 10–15 out of 15 on the sensitivity scale used in our study and so is particularly sensitive to fishing pressure). Such structure-building organisms have been shown to enhance habitat complexity and biodiversity and play an important role as settlement substrate for juveniles of many species [89]. At the same time, in places such as estuaries, overfishing is believed to have caused the ecological destruction of suspension feeder assemblages [123] and resulted in the shifting baseline conditions of fisheries, leading to inter-generational changes in perception of the state of the environment [124]. Although most of the decline has likely occurred before modern monitoring started [125], Lambert et al. [126] were able to predict an average of 17% (varying from 0to 66%) decrease in size and an average of 8% (0 to 34%) decrease in biomass of epifaunal communities as a result of fishing. Fishing may also be not detected (or have no impact) in areas with high natural disturbance [127], [128]. Nevertheless, the benthic communities currently observed are likely to also reflect the chronic pressure of bottom fishing on the seafloor [73].

In this study, abrasion resulting from bottom impacting fishing was found to be fully independent from PDS and had minimal relevance to community structure. The spatial distribution of abrasion reflects the distribution of the main commercial target species as well as equally important external drivers (such as habits, fuel costs, market demand and regulation) affecting fishers’ choice of fishing locations [72]. In the English Channel, where all sediment types are easily (and effectively) trawled, the abrasion distribution bears little to no relation to the local environment [129], [130], [131]. This study showed however that the TDI was negatively correlated to abrasion confirming both its usefulness to detect trawling impacts and confirming the extent of these impacts in our study area. Such relationship was already reported in other studies and the present study further confirms their findings and conclusions [127], [132], [133]. Shift in biological traits (body size, motility, prevalent feeding mode) in benthic communities in response to trawling is demonstrated in multiple ecosystems [109], [120], [134], [135], [136].

In the present study, we found that understanding of the relationship between PDS and TDI could be improved by using spatial distribution of abrasion as an additional forcing. This suggests that the departure of the TDI (that can be seen as an *in situ* sensitivity) from the PDS (natural potential sensitivity) could be explained to a large degree by fishing-induced abrasion. In the English Channel, sensitive benthic species distributions are shaped by both environmental processes and bottom contacting fisheries.

Finally, a PDS is a spatial model that strongly depends on the quality, reliability and resolution of input data. In this study, we have used 0.03 x 0.03 degrees resolution model grid interpolated from various sources with different original resolution (samples, models, climatological observations). Sediment grain size grid, for example, was calculated using observed mean grain size values from sediments samples assigned to each of the five sediment classes of a polygon-based sediment map, which inevitably led to loss of within-class variability. As a consequence, it may have weakened explanatory power of the Disturbance axis in our model. Temporal extent of climatological data (*e*.*g*. bottom temperature) may also have effect on the accuracy of the model by potential omission of long-term trends. However, operational implementation of any model is always dependent on input data availability and quality (*e*.*g*. [14], [25], [26]).

### Management implication

The maps produced using the PDS approach can have direct management applications. The disturbance map provides a baseline (disturbance of natural processes) against which additional disturbances from anthropogenic activities, especially the activities which add pressure on the bottom communities and habitats, may be measured. For example, areas with high natural disturbance level (corresponding to sandy areas in our study) are less likely to be affected by additional impacts on the seabed as local species are naturally well adapted to such conditions. Similarly, areas with low scope for growth are more susceptible to high fishing pressure as the time required to recover from removal will be long. Disturbance and scope for growth are closely linked to the concept of resistance (ability of a habitat to withstand a pressure without noticeable changes of biological or abiotic characteristics) and resilience (time needed for recovery when the pressure stopped), both considered as descriptors of the sensitivity [137]. According to Holling [7], disturbance and productivity descriptors were retained in the OSPAR convention and the MSFD to estimate the sensitivity of habitats. Sensitivity is now a key concept for ocean management and conservation [9], [10]. The PDS approach applied in this study predicts by design that the most sensitive habitats and communities are expected to combine low disturbance and low scope or growth.

However, in the light of the present results, the PDS may now no longer be fully observable in the field as a result of the ongoing human impacts of marine habitats. Natural marine habitats in trawled areas may have ceased to exist several decades ago and what can be observed nowadays might be a semi-natural habitat (similar to terrestrial grasslands) forced into a pseudo-climax by the intense and chronic use of bottom impacting fishing gear. In these conditions, it is difficult to *a posteriori* evaluate the effect of this human use, since reference points of anterior states are no longer available. Still the PDS may represent a good proxy of how natural processes alone would shape benthic communities and what the state of environment would be if it were not impacted by human activity.

In addition, the use of biological traits indicating trawling impact (such as TDI) in conjunction to spatial distribution of abrasion may reveal areas already seriously impacted, and those, still sheltered and sensitive, where conservation effort would be welcome. Similarly such knowledge may prevent fishing effort shift to sensitive areas following spatially limited fishing closure [138] and could inform marine spatial planning and ecosystem-based management [139]. Finally, the use of recurrent bottom trawl scientific surveys and TDI may constitute a good and cost effective monitoring technique within the MSFD to observe the effect of management measures for restoration of benthic habitat to less degraded states. The repeated evaluation of the distance (i.e. lack of correlation) between PDS and TDI would be a very helpful indicator in relation to the good environmental status objectives. As abrasion is reduced, the reduction of the distance between PDS and TDI would indicate a restoration of the benthic ecosystem composition towards a more “natural” state. In contrast, a fishery impacted state is indicated as long as the addition of abrasion, as a supplementary explanatory variable, improves the statistical relationship between TDI (observed *in situ*) and PDS (proxy of the natural state). We suggest that seabed management practices should aim for a reduction of the distance between TDI and PDS in areas where abrasion was shown to be negatively correlated with TDI.

## Conclusions

The PDS developed by Kostylev and Hannah [14] (i) avoids subdivisions of each physical layer into classes and the resulting map is a continuum where the gradients arise naturally from data layer compilation and (ii) models explanatory factors accepted by ecological community as main selective forces responsible for definition of biological traits of species. Considering its robustness, this method could be a useful tool for management [93], [140], [141] and for marine spatial planning [142], [143]. It could answer the need of the ecosystem-based approach, where process-driven mapping of representative habitat types would form a fundamental base for management [144]. Used in conjunction with TDI approaches applied to the most sensitive part of the benthic fauna, PDS may enable monitoring of management effort through evaluation of the distance that still separates impacted marine benthic habitats from the desired environmental status.

## Supporting information

S1 Table Taxon grouping and associated functional traits scoring: The maximum value of the regrouped species functional traits scoring was retained for each group, when taxonomic groupings were considered, the group indicator homogeneity was checked using standard deviation. If the SD of any given indicator for a group was above 1.5 and if the SD of the summed value over all 5 indicators was above 2.5, the group was removed from the analysis. This resulted in the removal of Bivalvia group which was found to be too heterogeneous as it accounted for 22 different initial taxa with very different functional.
(XLSX)

S2 Table 10 best results of the Bio-Env analysis. (XLSX)

S3 Table GLM results for TDI* ~ Risk + Abrasion, *log-transformed values. (XLSX)

S4 Table Spearman correlation matrix between the functional biological traits. (XLSX)

S1 File Calculations steps for estimating the friction velocity. (DOCX)
