# Supplementary material for: Process-driven and biological characterisation and mapping of seabed habitats sensitive to trawling
Source: PLoS One. 2017 Oct 5;12(10):e0184486. doi: 10.1371/journal.pone.0184486 (PMC5628812; doi:10.1371/journal.pone.0184486)
Supplement: S1 File — (DOCX) [file pone.0184486.s005.docx]

**Calculations for the « waves generated current » layer:**

Calculating steps:

- calcul(1)

$$\frac{\left( 4\times\pi^{2}\times\left[ depth \right] \right)}{\left( \left[ peak\_wave\_period \right]^{2}\times9.8 \right)}$$

- calcul(2)

$$\sqrt{\frac{(e^{calcul\left( 1 \right)}- e^{-calcul\left( 1 \right) )}}{(e^{calcul\left( 1 \right)}+ e^{-calcul(1)})}}$$

- calcul(3)

$$\frac{(9.8\times\left[ peak_{wave_{period}} \right]^{2})}{(2\times\pi)}$$

- calcul(4)

$$calcul(2)\times calcul(3)$$

- calcul(5)

$$\frac{(2\times\pi)}{calcul(4)}$$

- calcul(6)

$$\frac{(e^{\left( calcul\left( 5 \right)\times\left[ depth \right] \right)}- e^{(-calcul(5)\times\left[ depth \right]})}{2}$$

- calcul(7) = final grid named “Waves generated current”

$$\frac{(\pi\times\frac{[wave\_height]}{[peak\_wave\_period]}}{calcul(6)}$$
